# Transcriptome analysis of pecan seeds at different developing stages and identification of key genes involved in lipid metabolism

**DOI:** 10.1371/journal.pone.0195913

**Published:** 2018-04-25

**Authors:** Zheng Xu, Jun Ni, Faheem Afzal Shah, Qiaojian Wang, Zhaocheng Wang, Lifang Wu, Songling Fu

**Affiliations:** 1 College of Forestry and Landscape Architecture, Anhui Agricultural University, Hefei, Anhui, China; 2 Key laboratory of High Magnetic Field and Ion Beam Physical Biology, Hefei Institutes of Physical Science, Chinese Academy of Sciences, Hefei, Anhui, PR China; Huazhong University of Science and Technology, CHINA

## Abstract

Pecan is an economically important nut crop tree due to its unique texture and flavor properties. The pecan seed is rich of unsaturated fatty acid and protein. However, little is known about the molecular mechanisms of the biosynthesis of fatty acids in the developing seeds. In this study, transcriptome sequencing of the developing seeds was performed using Illumina sequencing technology. Pecan seed embryos at different developmental stages were collected and sequenced. The transcriptomes of pecan seeds at two key developing stages (PA, the initial stage and PS, the fast oil accumulation stage) were also compared. A total of 82,155 unigenes, with an average length of 1,198 bp from seven independent libraries were generated. After functional annotations, we detected approximately 55,854 CDS, among which, 2,807 were Transcription Factor (TF) coding unigenes. Further, there were 13,325 unigenes that showed a 2-fold or greater expression difference between the two groups of libraries (two developmental stages). After transcriptome analysis, we identified abundant unigenes that could be involved in fatty acid biosynthesis, degradation and some other aspects of seed development in pecan. This study presents a comprehensive dataset of transcriptomic changes during the seed development of pecan. It provides insights in understanding the molecular mechanisms responsible for fatty acid biosynthesis in the seed development. The identification of functional genes will also be useful for the molecular breeding work of pecan.

## Introduction

*Carya illinoinensis*, also known as pecan, and is originated from the North America [1]. Pecan is now an economically important tree nut crop all over the world, the seed of which has unique texture and flavor properties [2, 3]. The basic nutritional composition of pecan includes fatty acids, protein, phytochemicals and some other bioactive compounds [4]. Pecan nuts have been shown to contain very high levels of antioxidants, thus its consumption has been associated with several health benefits, including improved serum lipid profile [5]. The seed kernel contains a high level of oil (70–79%), which is mainly composed of unsaturated fatty acids, such as oleic acid (60–70%) and linoleic acid (19–30%) [6]. Pecan oil is very low in saturated fatty acids (<9%), and the concentration of monounsaturated fatty acids is higher than olive oil [6]. All these characteristics suggested that pecan oil is ideal for dietary purposes.

Fatty acids perform multiple functions in plants, including serving as important energy reserves, membrane components, signaling molecules and even playing important roles in plant defenses [7, 8]. Lipid biosynthesis of nuts depends on the spatial and temporal activity of many gene products that are involved in de novo biosynthesis of fatty acid, synthesis of triacylglycerol (TAG) and oil body formation [9–11]. The development of pecan seeds lasts for a long period of time, from May to October. The structure and chemical compounds of the seeds changed significantly, such as embryo and endosperm development and fatty acids biosynthesis at different developing stages. The lipid in the pecan seeds accumulated significantly during the late stages of seed development. However, how genes control the initiation and de novo biosynthesis of the lipid in pecan remains largely unknown. Identification of key genes controlling quantitative features of fatty acids biosynthesis in pecan is of significant importance.

High-throughput sequencing technologies are efficient and data-rich [12, 13], which enables the global gene expression analyses of seed development at different developing stages. The transcriptomic information is lacking for pecan. In this research, we are interested in identification of genes involved in seed development, especially fatty acid biosynthesis. The transcriptome sequencing was first carried out by using seeds collected at different developing stages. The comparative transcriptomic analyses of seeds, separately at two developing stages (before and after oil synthesis) were further investigated. In this study, a total of 82,155 unigenes were generated from the seed transcriptome of pecan, among which 13,325 unigenes showed a 2-fold or greater expression difference between the two seed developing stages. The identification of the differentially expressed genes between these two developmental phases has allowed us to discover most genes that are related to lipid metabolism. The transcriptome data presented in this work provide useful information of pecan seed development in the transcriptomic and molecular levels.

## Materials and methods

### Plant materials

The pecan nuts were collected from the 16-year-old pecan cultivar “Annong3” trees in Anhui, China. Each tree received standard agronomic practices. After pollination, the samples were collected every two weeks during the whole development period from June to October. After removing the pericarp and seed coat, the samples were frozen in liquid nitrogen and then stored at -80°C.

### Lipid analysis

Seeds harvested at different developing stages were dried at 105°C to a constant weight. Total lipids were measured by Soxhlet extraction method [14]. The pecan embryos were ground using a Philips mill. The power was packed in a thimble and soaked with petroleum ether for 1.5h. After oil extraction, the oil was dried at 105°C for 5h to remove the water and petroleum ether. Seed oil content was calculated on the basis of the weight of oil and dry seeds.

### RNA isolation and sequencing

A total of seven samples were collected for transcriptome sampling: Mixed sample of pecan seeds collected from different developmental stages (PM), and seeds at initial stage (PA, 85–95 DAP), and fast oil accumulation stage (PS, 125–135 DAP). Samples at initial stage and fast oil accumulation stage were prepared in triplicates. RNA isolation was performed using the Biozol Plant RNA Extraction Kit, as previously described. RNA quantity and quality were assessed using a NanoDrop 2000c Spectrophotometer (Thermo Scientific, Wilmington, DE, USA) and agarose gel electrophoresis, respectively. The RNA samples were further assessed using the Agilent Bioanalyzer 2100 system (Agilent Technologies, CA, USA). The mRNA was enriched from total RNA using oligo (dT) magnetic beads and fragmented into approximately 200-bp fragments. The cDNA was synthesized using a random hexamer primer and purified with magnetic beads. After the end reparation and 3ˈ end single nucleotide acid addition, the adaptors were ligated to the fragments. The fragments were enriched through PCR amplification and purified using magnetic beads. The libraries were assessed using the Agilent 2100 Bioanalyzer and quantified using the ABI StepOnePlus Real-Time PCR System. The samples were sequenced on an Illumina HiSeq 2000 with paired ends (BGI Tech, Shenzhen, China).

### De novo assembly of transcriptome and abundance estimation

Low-quality reads with Phred scores < 20 were trimmed using Fastq_clean [15], and the data quality was assessed using FASTQC [16]. The filtered reads were assembled using Trinity (version 2.0.6) with default parameters [17, 18]. The paired-end reads from each library were mapped to de novo assemblies using bowtie (version 1.1.1) [19]. The transcript abundance was estimated using Corset (version 1.03) [20]. The count data generated from Corset were processed using the edgeR package [21]. Transcripts with less than one count per million reads (CPM) for at least three libraries were removed, and the remaining data were used for the next analysis. A matrix was constructed using the single factor style. Effective library sizes were determined using the trimmed mean of M values (TMM) normalization method. The common dispersion and tag wise dispersion were estimated using the quantile-adjusted conditional maximum likelihood (qCML) method. The exact test was performed to compute the expression of genes between the treatment and mock groups. Raw P values were adjusted for multiple testing using a false discovery rate (FDR) [22]. Genes with an FDR of less than 0.05 and fold-changes greater than 2 were regarded as DEGs. GO analysis of the DEGs and pathways were processed using DAVID [23]. Hierarchical clustering of the genes was performed using the pheatmap R package (version 1.0.7) [24].

### Quantitative real-time PCR analysis

For each sample, 1 μg total RNA was used for cDNA synthesis using the PrimeScript Kit (TaKaRa Biotechnology, Dalian, China). TaKaRa SYBR Premix Ex Taq II (TaKaRa Biotechnology, Dalian, China) was used for qPCR. qPCR was performed on the Roche Light Cycler 96 System (Roche, Swiss). Each sample contains three independent biological replicates. The information of the primers used in the qPCR analysis was listed in S1 Table.

## Results and discussion

### Sample collection and Illumina paired-end transcriptome sequencing

**Fig 1:**
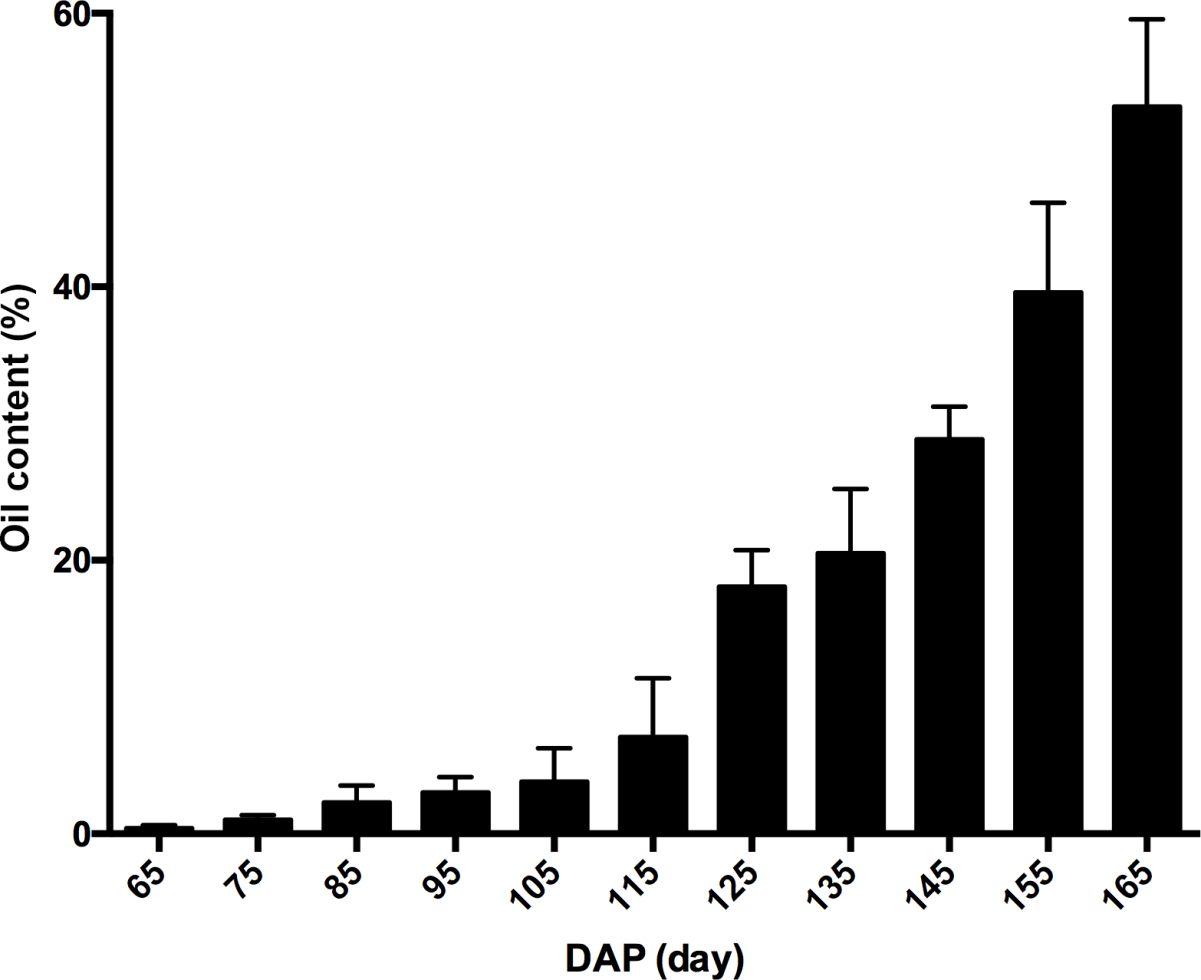
Oil content was determined at different developing stages of pecan seed. Since 65 days after pollination, the samples were collected for oil determination every 10 days, until the pecan seed was ripen (n = 3). Values are mean±SE.

The dynamic fatty acid contents of developing pecan seeds collected from 65 DAP and 165 DAP (every two weeks) were characterized. The results showed that the fast lipid accumulation started at 95 DAP and lasted to 125 DAP. Prior to 85 DAP (before August), low level of lipid can be detected was less than 0.05% (Fig 1). It increased slowly after 95 DAP and reached 23.6% at 125 DAP, resulting in 40.95% increment rate in average. Since 95 DAP, the ratio of oil to fat increased significantly and the unsaturated fat started to accumulate. To obtain the information of genes involved in seed development of pecan, the embryos at different developmental stages was collected and then used for the following cDNA library construction. The other six cDNA libraries were constructed from two stages of developing seeds (i.e., the initiate stage, PA and the fast oil accumulation stage, PS; each stage contains three independent biological replicates) and sequenced by Illumina high-throughput sequencing platform. After filtering the low-quality, adaptor-polluted and high content of unknown base, a total of 82,155 unigenes were assembled. The mean length of these unigenes was 1,198 bp and GC content was 42.75% (Table 1). A scatter plot was produced to show the transcript size distributions. We then annotated these unigenes with 7 functional databases and 56,634 (NR: 68.94%), 56,391 (NT: 68.64%), 39,683 (Swissprot: 48.30%), 22,949 (COG: 27.93%), 43,881 (KEGG: 53.41%), 32,499 (GO: 39.56%), and 39,613 (Interpro: 48.22%) were annotated (S2 Table). We detected 55,854 CDS and predicted 2,807 transcription factors (TF) coding unigenes. It was also found that 699 SSR distribute on 17,087 unigenes. The identification of unigenes from pecan seeds at different developmental stages could provide a basis for future research, such as transcriptome analysis, gene cloning and transgenic studies.

**Table 1:** Summary statistics of clean reads in the cDNA library of pecan seeds at different developing stages (PM) and six libraries of pecan seeds at two developing stages (PA and PS).

Sample	Total number	Total length	Mean length	N50	N70	N90	GC (%)
PM	69051	72895611	1055	1773	1136	428	42.58
PA-1	40065	44845730	1119	1767	1183	492	43.8
PA-2	42733	46468170	1087	1736	1150	467	43.67
PA-3	48074	48539950	1009	1652	1055	413	43.45
PS-1	38472	38294243	995	1608	1030	409	43.91
PS-2	39765	39673734	997	1611	1039	409	43.79
PS-3	38847	38795394	998	1603	1038	412	43.87

N50: a weighted median statistic that 50% of the Total Length is contained in transcripts great than or equal to this value. GC (%): the percentage of G and C bases in all transcripts.

### Functional characterization of the unigenes of pecan by Gene Ontology, Clusters of Orthologous Groups and KEGG pathway analysis

**Fig 2:**
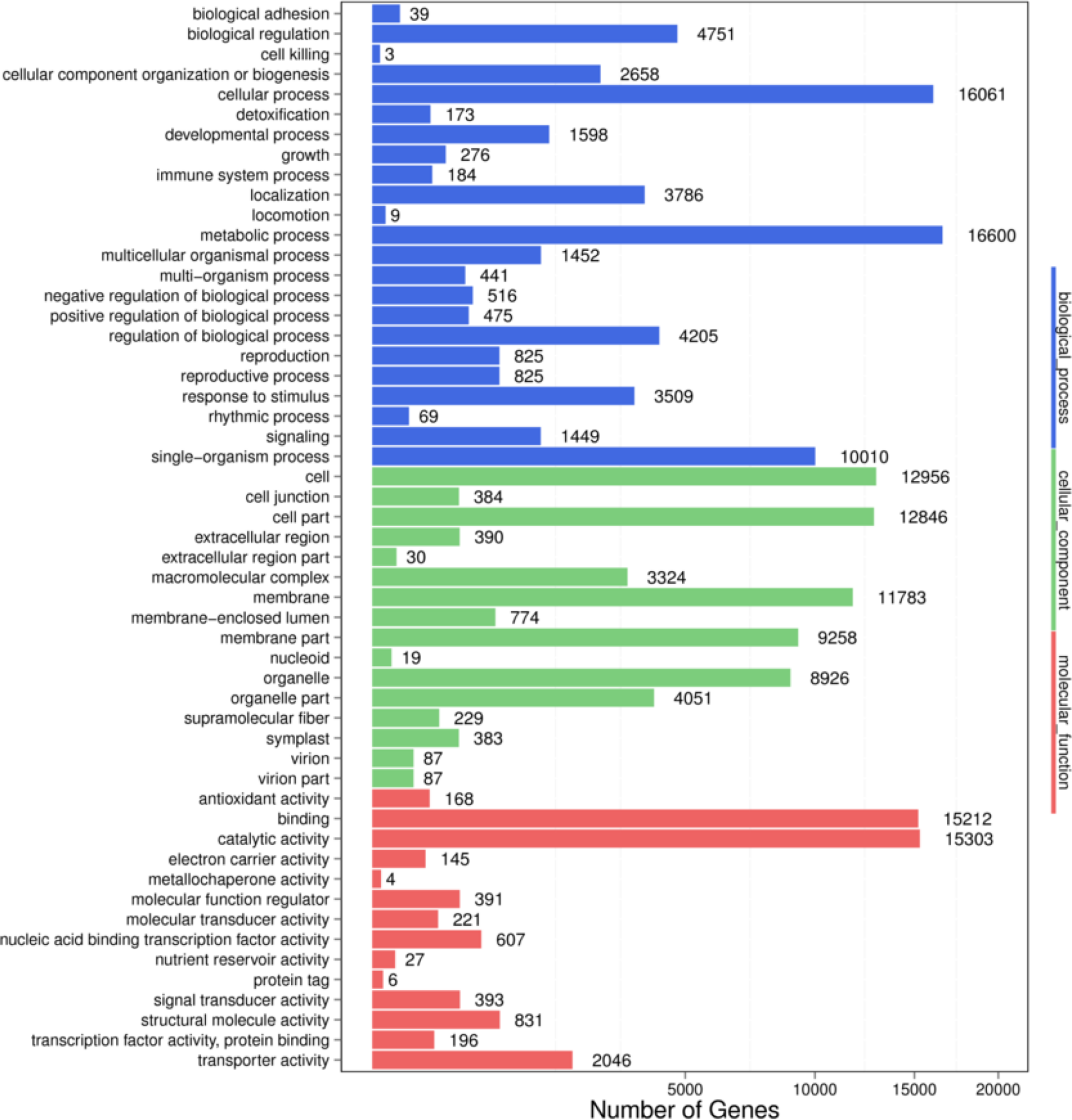
Functional distribution of GO annotation of all assembled unigenes. The Unigenes were assigned to three main categories: biological processes, cellular components, and molecular functions. X axis represents the number of unigenes. Y axis represents the Gene Ontology functional categories.

Gene Ontology assignments were used to classify functions of the predicted pecan seed unigenes. 32,499 (39.56%) unigenes were categorized into 53 functional groups under three main divisions (biological process, cellular components, and molecular functions) (Fig 2). In the biological process, cellular process (16061 unigenes), metabolic process (16600 unigenes) and single-organism process (10010 unigenes) are most abundant groups, followed by biological regulation, localization and regulation of biological process. In the cellular component, cell (12956 unigenes), cell part (12846 unigenes), membrane (11783 unigenes), membrane part (9258 unigenes), and organelle (8926 unigenes) were the predominant groups, followed by macromolecular complex (3324 unigenes), organelle part (4051 unigenes). In the molecular function, binding (15212 unigenes) and catalytic activity (15303 unigenes) were the predominant groups, followed by transporter activity (2046 unigenes).

**Fig 3:**
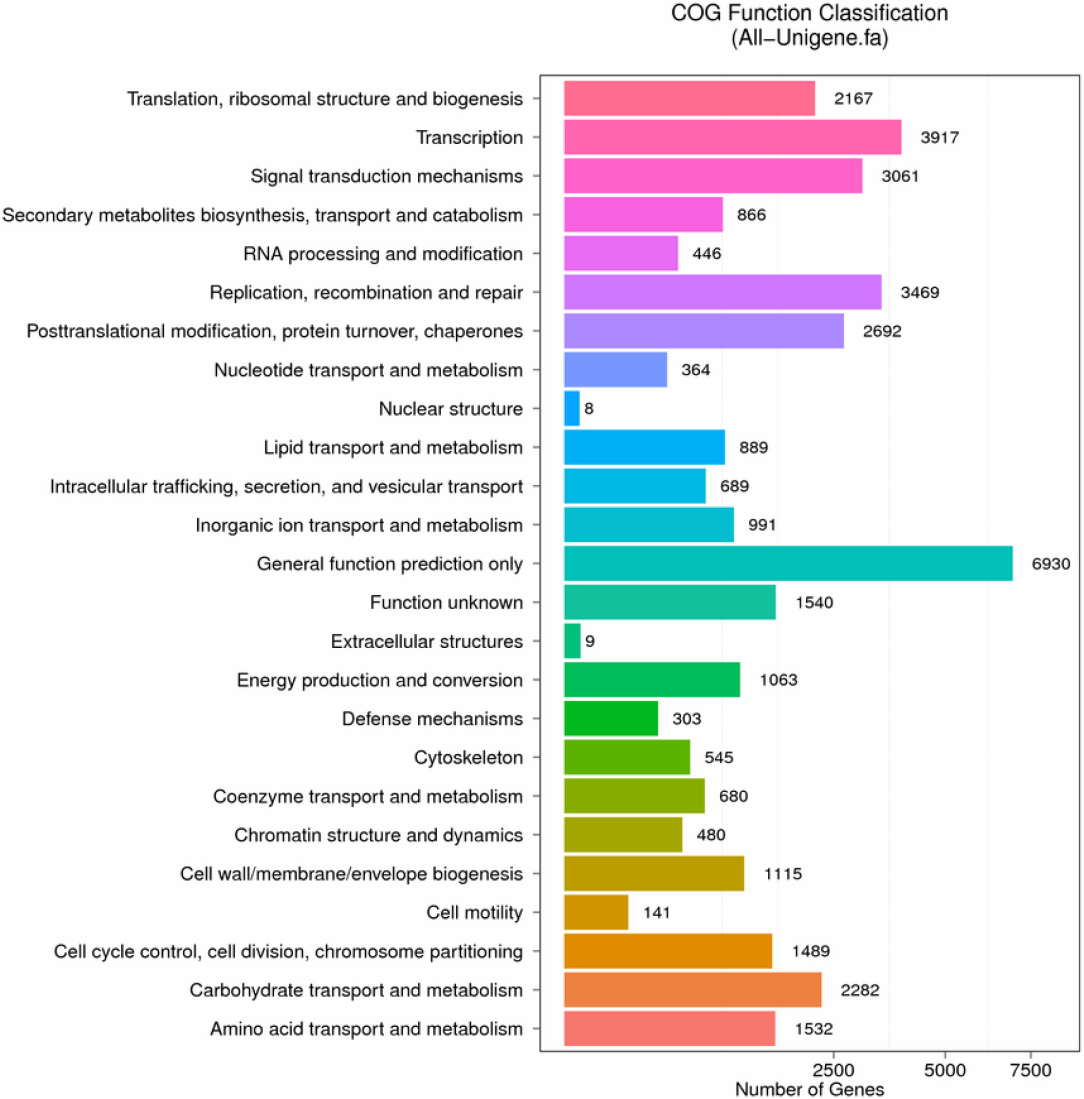
Clusters of Orthologous Groups (COG) classification of all assembled unigenes. A total of 22,949 unigenes were classified into 25 functional categories. X axis represents the number of unigenes. Y axis represents the COG functional categories.

**Fig 4:**
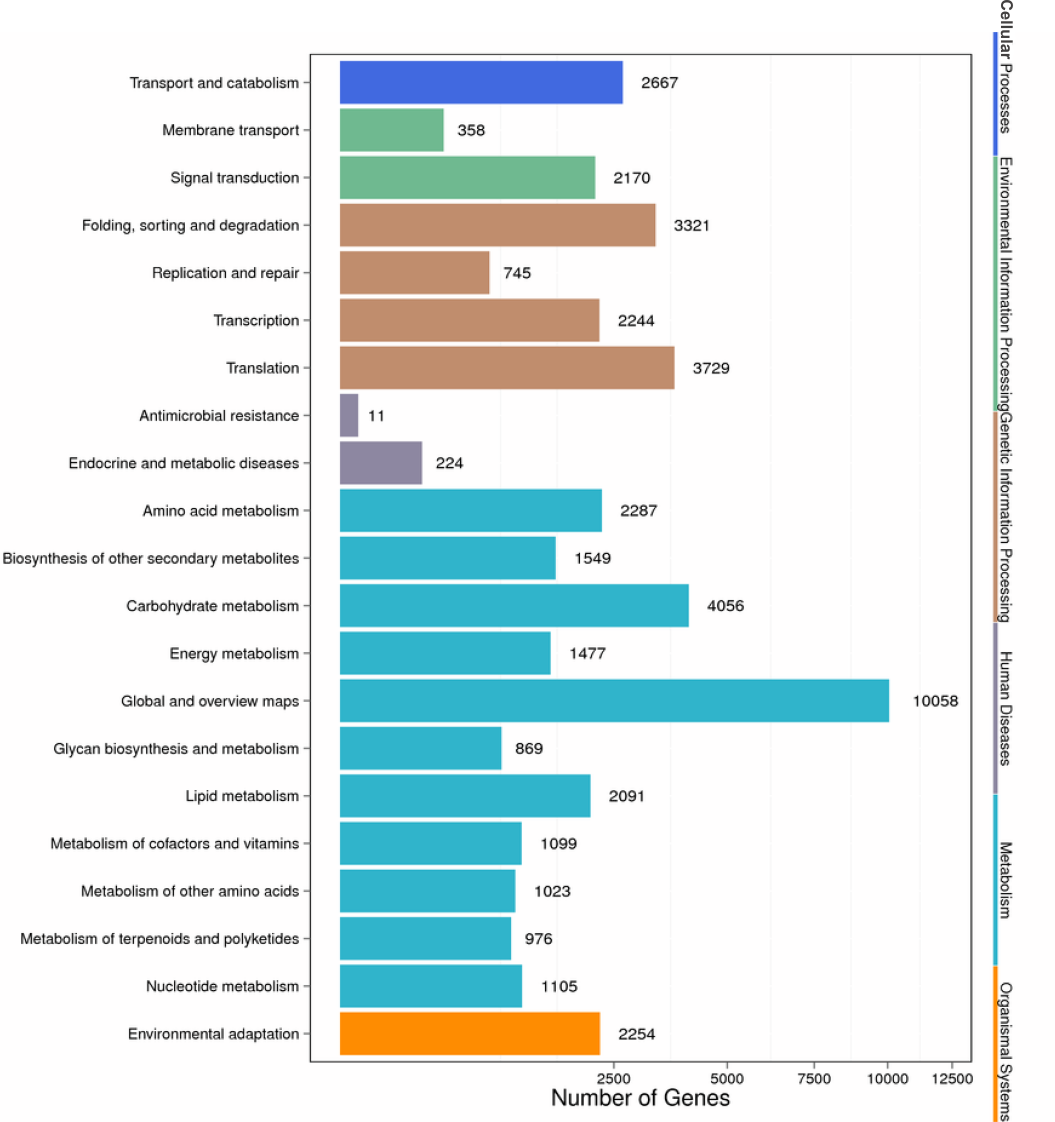
Functional distribution of KEGG annotation of the assembled unigenes. X axis represents the number of Unigenes. Y axis represents the KEGG functional categories.

The unigenes of pecan seed were also compared with the Clusters of Orthologous Groups and KEGG databases for further functional prediction. By COG analysis, a total of 22,949 unigenes were annotated and classified into 25 groups (Fig 3). The most abundant groups were “General function prediction only” (6930), “Transcription” (3917), “Replication, recombination and repair” (3469) and “Signal transduction mechanisms” (3061). Among these 25 groups, abundant unigenes were also classified into “Lipid transport and metabolism”, “Energy production and conversion”, and “Carbohydrate transport and metabolism”, which was closely related to seed oil biosynthesis. Pathway-based analysis of the seed unigenes of pecan by KEGG can further our understanding of the gene functions. A total of 43,881 unigenes were classified into 21 groups (Fig 4). The predominant groups included, “Carbohydrate metabolism” (4056), “Translation” (3729), “Folding, sorting and degradation” (3321), “Lipid metabolism” (2091), “Amino acid metabolism” (2287) and “Transport and catabolism” (2667). Conclusively, abundant unigenes or pathways annotated by GO, COG and KEGG analysis, were closely linked to the changes in oil content and composition that take place during the pecan seed ripening. The information of the identified unigenes that could be involved in the lipid biosynthesis would be very helpful in the following research.

### Identification of transcription factors during pecan seed development

**Fig 5:**
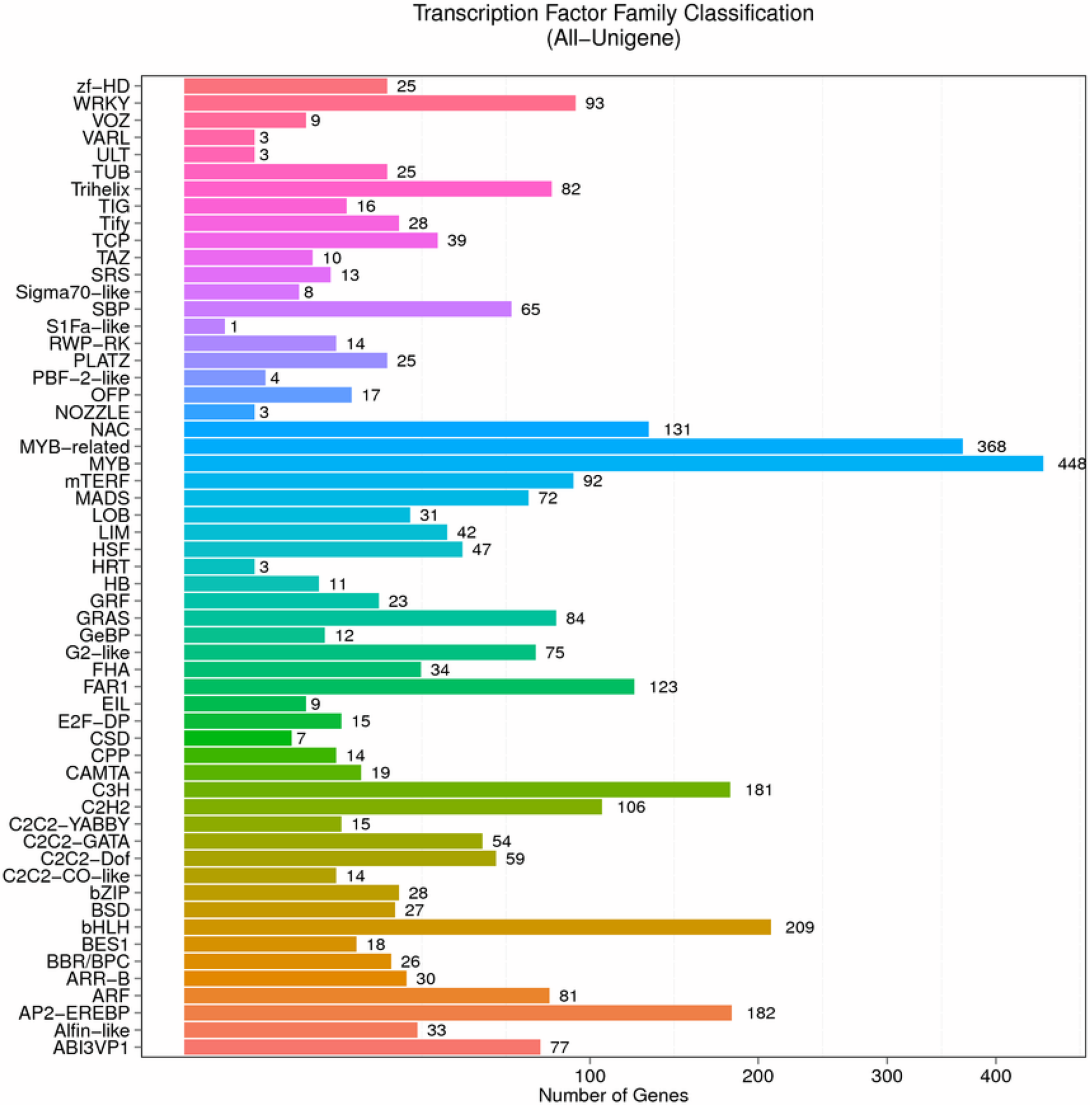
Transcription factor family classifications of the unigenes throughout the whole developing stages of pecan seed. 2,807 transcription factors were predicted and classified into 57 transcription factor families.

Transcription factors are powerful regulators in controlling gene expression almost in every aspect of plant growth and development [25–27]. The lipid biosynthesis is also controlled by many key transcription factors, such as *WRINKLED1* (*WRI1*) [28], *LEAFY COTYLEDON 1* (*LEC1*) [29], *LEAFY COTYLEDON 2* (*LEC2*) [30], *FUSCA3* (*FUS3*) [31] and *ABSCISIC ACID INSENSITIVE 3* (*ABI3*) [32]. After annotation, 2,807 were predicted to be transcription factor (TF) coding unigenes. The predicated transcription factors were further classified into 57 types (Fig 5). The most abundant groups were *MYB* (448), *MYB*-related (368), *bHLH* (209), *C3H* (181), *NAC* (131) and *FAR1* (123). The results suggested that these types of transcription factors played important role during pecan seed development, including oil biosynthesis and accumulation.

### Gene expression profile at two developmental stages

**Fig 6:**
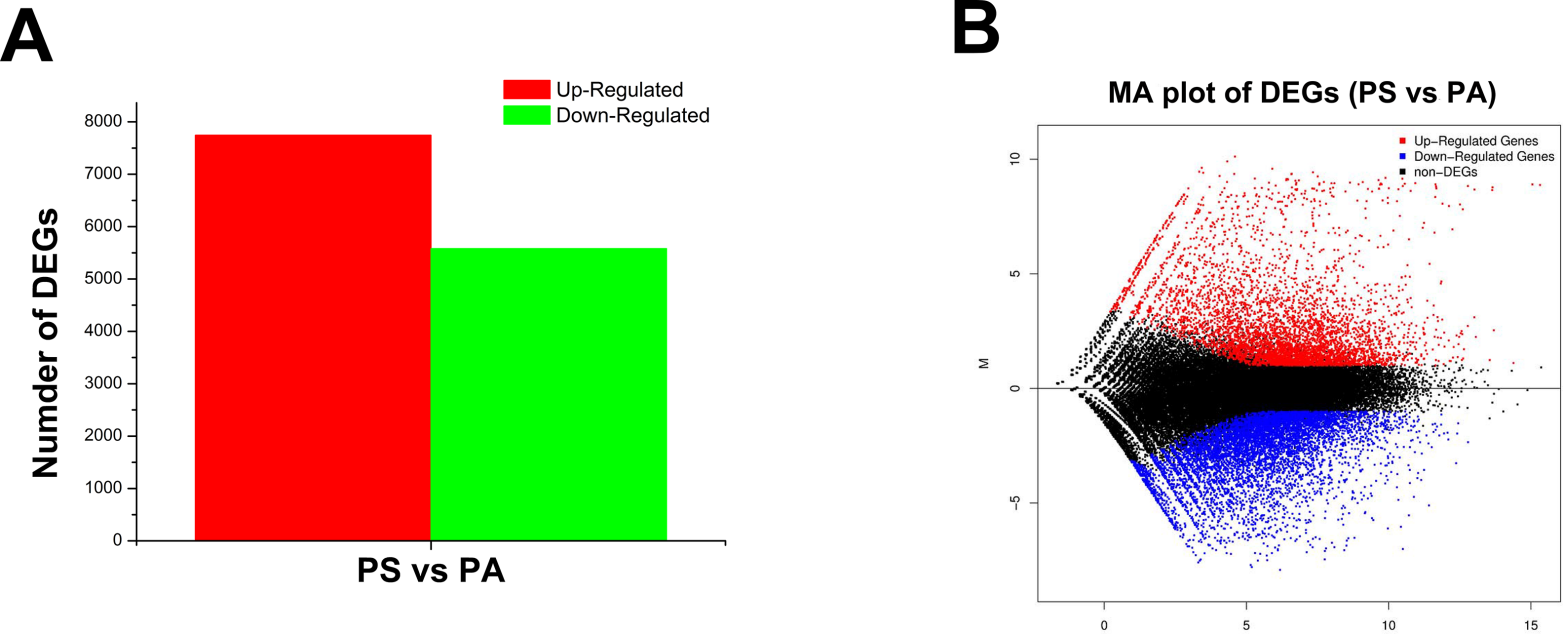
Comparison of gene expression between the fast oil accumulation stage (PS, 125 DAP) and the initial stage (PA, 95 DAP). (A) An average of 5580 and 7745 unigenes were separately up-, and down-regulated between PS and PA. (B) MA plot of the unigenes between PS and PA.

The transcriptome of pecan seed at fast lipid accumulation stage (PS) was compared with that at initial stage (PA) to identify genes involved in the lipid biosynthesis. A general picture of the differentially expressed genes was plotted for PS library versus PA library. A total of 13,325 unigenes showed ≥ 2-fold expression change between PS and PA libraries. Among these, 5580 unigenes were up-regulated, and 7745 were down-regulated (Fig 6). The 30 most abundant unigenes in PA liberary were selected for further functional analysis. We noticed that many unigenes are cell wall structural constituent related, such as “proline-rich protein” [33] (CL328. Contig2_All, CL1580.Contig5_All, CL328.Contig1_All, CL1580.Contig3_All, CL328.Contig3_All, and CL1580.Contig7_All), “xyloglycan endotransglycosylase” (CL4283.Contig2_All, CL4283.Contig1_All and CL6948.Contig2_All) (Table 2). The high expression of cell wall constituent-related genes could be consistent with the fast cell growth and amplification of the seed embryo at the early development stages. Among the most abundantly expressed genes in PS libraries, seven unigenes (Unigene7604_All, CL2857.Contig3_All, CL10064.Contig2_All, Unigene1979_All, CL2857.Contig1_All, Unigene3155_All, Unigene19032_All), were identified to encode oleosins, which are an important part of oil bodies. Further, four unigenes (Unigene5477_All, CL3568.Contig2_All, CL9901.Contig1_All, and CL9901.Contig2_All) encoding storage proteins, were also found to be abundantly expressed at the PS stage. DIACYLGLYCEROL ACYLTRANSFERASE 1 (DGAT1) (Unigene16582_All) which is a rate-limiting enzyme controlling lipid biosynthesis, was highly expressed in the PS stage (Table 3). Taken together, those unigenes abundantly expressed in the PS library could be closely correlated with the fast protein and lipid accumulation in the pecan seed.

**Table 2:** Most highly expressed unigenes in the initial stage (PA) of pecan seed development.

Gene ID	Gene Product	Length	PA-Mean (RPKM)	PS-Mean (RPKM)
Unigene19077_All	Glycine-rich cell wall structural protein	114	4729.04	15.08333333
Unigene3355_All	Metallothionein type 2	78	3209.796667	3484.78
CL3605.Contig2_All	Uncharacterized protein	152	3167.13	3802.766667
Unigene12186_All	Lipid-transfer protein DIR1	91	3105.726667	194.6166667
CL4283.Contig2_All	Xyloglucan endotransglucosylase	295	2092.356667	29.53
CL4054.Contig1_All	DNAJ	417	1976.833333	612.68
Unigene163_All	Heat shock protein 70	471	1936.653333	755.24
Unigene19742_All	UBQ10	193	1850.91	1366.03
CL4184.Contig1_All	Eukaryotic aspartyl protease	427	1395.003333	350.76
CL328.Contig2_All	14 kDa proline-rich protein	153	1374.89	69.64333333
CL4612.Contig1_All	Unkown	56	1371.193333	5.42
CL1580.Contig5_All	36.4 kDa proline-rich protein	462	1349.293333	832.8333333
CL80.Contig1_All	Ripening-related protein-like	156	1312.823333	458.8433333
CL328.Contig1_All	14 kDa proline-rich protein	158	1304.696667	48.24666667
CL328.Contig7_All	Lipid-transfer protein	178	1227.796667	48.11
Unigene21221_All	Formin-like protein	77	1196.253333	8.676666667
CL1329.Contig2_All	Beta-glucosidase BoGH3B-like	621	1165.523333	112.59
CL1580.Contig3_All	36.4 kDa proline-rich protein isoform	466	1155.63	468.45
Unigene21331_All	Flavanone 3-hydroxylase	343	1147.283333	14.48
CL328.Contig3_All	14 kDa proline-rich protein	168	1137.25	52.26666667
CL4283.Contig1_All	Xyloglucan endotransglucosylase	293	1134.43	33.63666667
Unigene16691_All	Peroxidase 42	331	1126.963333	1434.876667
Unigene3610_All	Unkown	113	1119.24	3.06
Unigene14929_All	UBC7	199	997.08	174.2233333
CL6948.Contig2_All	Xyloglucan endotransglucosylase	284	994.48	7.89
CL1580.Contig7_All	Proline-rich protein-like isoform	550	975.5	519.5233333
CL4612.Contig2_All	Unknown	56	968.1666667	3.683333333
CL4054.Contig2_All	DNAJ protein homolog	417	935.0833333	200.74
Unigene7612_All	Auxin-repressed protein	117	923.27	46.13333333
Unigene21319_All	Unknown	169	917.8233333	130.6

**Table 3:** Most highly expressed unigenes in the fast oil accumulation stage (PS) of pecan seed development.

Gene ID	Gene product	Length	PS-Mean (RPKM)	PA-Mean (RPKM)
Unigene5477_All	11S legumin protein	501	46037.01667	157.2366667
CL3568.Contig2_All	CRA1, 12S seed storage protein	488	36762.03333	124.0266667
Unigene679_All	Allergen I1	143	20448.71	81.6
CL4551.Contig1_All	GRPF1	92	19656.52	69.24
CL10249.Contig1_All	7S vicilin	793	9682.446667	35.68
CL10249.Contig2_All	7S vicilin	733	8689.473333	34.58
Unigene7604_All	Oleosin	160	6685.183333	22.37
CL4551.Contig2_All	GRPF1	58	6575.096667	25.03666667
CL9901.Contig1_All	11S globulin seed storage protein	464	5134.46	31.70333333
Unigene42036_All	HSP20-like chaperones	159	4956.676667	17.67333333
CL9901.Contig2_All	11S globulin seed storage protein	464	4755.606667	34.08
Unigene21014_All	heat shock protein	159	4554.18	22.74
CL2857.Contig3_All	Oleosin	139	4112.786667	13.87666667
CL10064.Contig2_All	Oleosin	138	3848.833333	13.79333333
CL3605.Contig2_All	Major latex allergen Hev b 5	152	3802.766667	3167.13
Unigene1979_All	Oleosin	139	3721.266667	12.24333333
Unigene933_All	Defensin-like protein 1	75	3672.083333	14.58666667
Unigene3355_All	Metallothionein type 2	78	3484.78	3209.796667
CL307.Contig2_All	Metallothionein-like protein	78	3346.99	42.1
Unigene3509_All	Aquaporin TIP3-2	255	3306.03	10.87
Unigene9192_All	Uncharacterized protein	121	3240.87	8.97
Unigene7887_All	Defensin-like protein 1	74	3147.673333	8.746666667
Unigene7899_All	48-kDa glycoprotein precursor	477	3122.153333	19.23
CL2857.Contig1_All	Oleosin	139	2749.76	15.62666667
Unigene3155_All	Oleosin	152	2100.493333	6.96
CL7893.Contig1_All	Thiamine thiazole synthase	355	1951.773333	574.6266667
Unigene9187_All	Uncharacterized protein	120	1727.156667	5.203333333
Unigene9200_All	Centromere-associated protein	130	1723.143333	7.083333333
Unigene19032_All	Oleosin	161	1705.956667	9.503333333
Unigene16582_All	DGAT1	453	1584.443333	19.60666667

**Fig 7:**
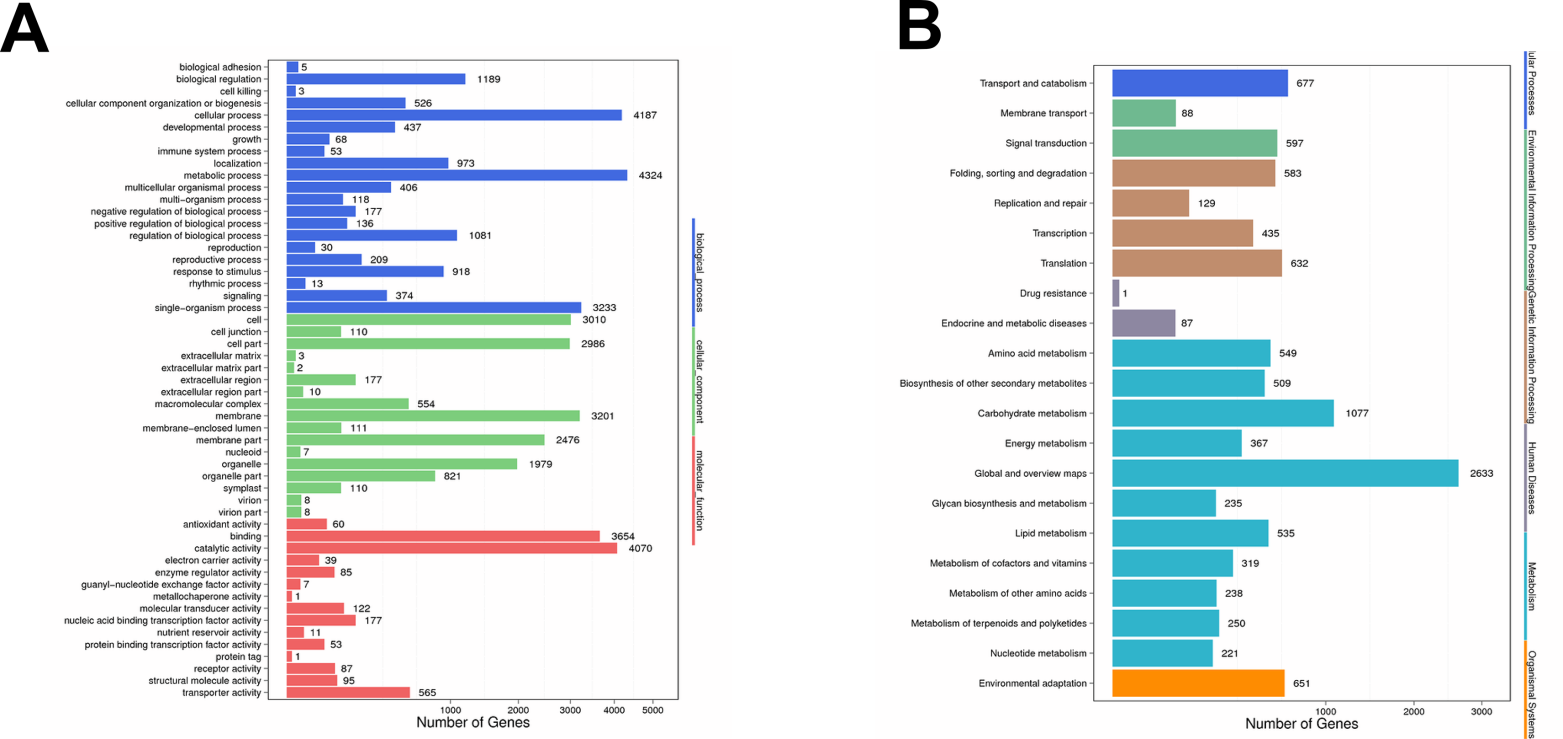
Gene Ontology (GO) functional and KEGG pathway classification of unigenes differentially expressed between PS and PA stages of pecan seed development. Unigenes were classified into three main categories: biological processes, molecular function and cellular component for GO analysis, and into six main categories: cellular processes, environmental information processing, genetic information processing, human diseases, metabolism and organismal systems. X axis represents the number of Unigenes. Y axis represents the GO or KEGG functional categories.

**Fig 8:**
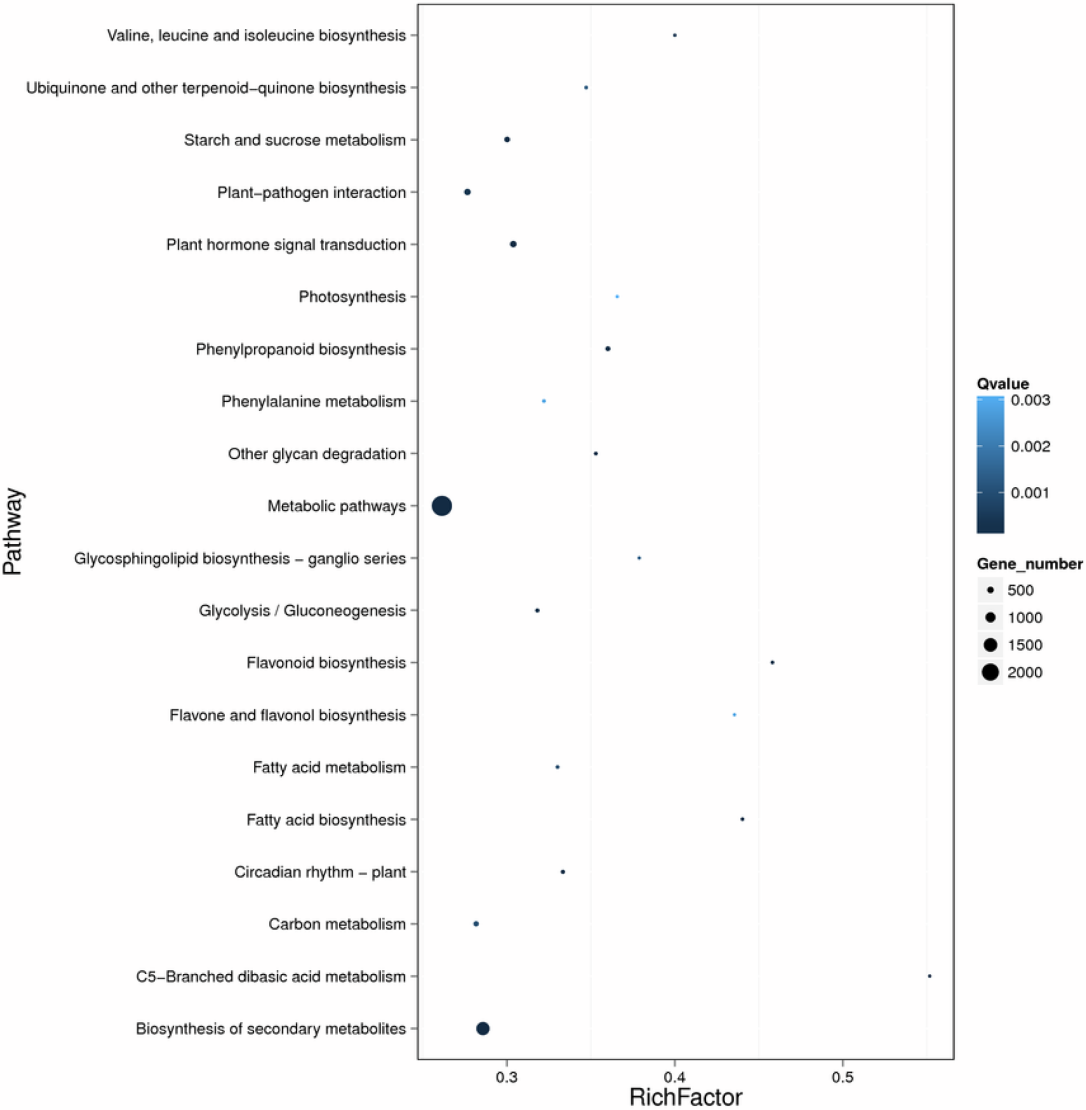
Pathway functional enrichment of the unigenes differentially expressed between PS and PA stages of pecan seed development. X axis represents the enrichment factor. Y axis represents pathway name. Coloring indicates q value (high: white, low: blue), the lower q value indicates the more significant enriched. Point size indicates DEG number (more: big, less: small).

To further identify the functions of the differentially expressed unigenes (DEGs) between PA and PS libraries. Gene Ontology analysis was carried out on the 13,325 unigenes. In the “biological process” category, these unigenes were classified into 21 groups (Fig 7). The predominant groups are “metabolic process”, “cellular process” and “single-organism process”, followed by “biological regulation”, “regulation of biological process” and “localization”. In the “cellular component” category, the most presented groups were “membrane”, “cell”, “cell part”, “membrane part”, and “organelle”. These DEGs were divided into 15 groups according to their molecular functions; the predominant groups were “binding” and “catalytic activity”. Further, the KEGG pathway classification was carried out for the functional enrichment for the unigenes. The DEGs were classified into 21 groups, half of which were belonging to the “metabolism” category (Fig 8). The most abundant groups, including carbohydrate metabolism, amino acid metabolism, lipid metabolism and energy metabolism, were closely related to seed oil and storage protein biosynthesis. After pathway functional enrichment, we noticed that abundant DEGs were included in “metabolic pathways”, “biosynthesis of secondary metabolites”, “starch and sucrose metabolism”, “phenylpropanoid biosynthesis”, and “fatty acid metabolism”, which were closely related to the fast lipid and storage protein accumulation at the fast oil accumulation stage. The pathway analysis of the DEGs between PS and PA libraries could provide valuable information for the following research on the seed development of pecan.

### Identification of unigenes related to the fatty acid biosynthesis

Pecan seeds can accumulate considerable amounts of unsaturated fatty acids at the late developing stages. Thus, the comparison of the transcriptome libraries of pecan seeds at the initial stage and the fast oil accumulation stage may help us to identify the key regulators involved in the regulation of fatty acids biosynthesis. Based on the functional annotation and GO analysis of the DEGs, we summarized the expression levels of DEGs that could be involved in fatty acid and TAG biosynthesis. For fatty acid biosynthesis, 81 unigenes were identified, including 6 genes coding acetyle-CoA carboxylase (ACCase), 2 for enoyl-ACP reductase (EAR), 10 unigenes encoding 3-ketoacyl-ACP synthases (KAS) (2 for KASI, 5 for KASII, 2 for KASIII and 1 for KASIV, respectively), 3 unigenes encoding fatty acid synthase (2 for enoyl-ACP dehydrase EAR and 1 for ketoacyl-ACP reductase KAR) (Table 4). The RPKM values showed that these genes involved in the de novo FA biosynthesis were up-regulated in the fast oil accumulation stage, which is in accord with the oil biosynthesis undergoing at this period. In addition, three unigenes encoding thioesterases, which can produce free FAs (2 for acyl-ACP thioesterase A FATA and one for acyl-ACP thioesterase B FATB), 15 unigenes encoding long-chain acyl-CoA synthetases, which catalyzes esterification of free FAs to CoA, and 6 unigenes encoding acyl-CoA binding proteins (ACBP, acyl-CoAs transportors). The transcriptome results showed that these unigenes were up-regulated at the fast oil accumulation stage, indicating their pivotal roles in FA synthesis in pecan seeds. For the formation of unsaturated FAs, 10 unigenes encoding fatty acid desaturase were identified, including 2 unigenes encoding stearoyl-ACP desaturase (SAD), which removes two hydrogene atoms from stearic acid to form oleic acid, 8 unigenes encoding oleate desaturase (5 for FAD2, 1 for FAD3, and 2 for FAD7), which can remove two hydrogene atoms from oleic acid to form linoleic acid. Oleic acid, which is directly catalyzed by the fatty acid desaturases, is the predominant unsaturated fatty acid. The high expression level of the fatty acid desaturases at the fast oil accumulation stages could lead to fast biosynthesis of the unsaturated fatty acids in the pecan seeds.

**Table 4:** Unigenes related to fatty acid biosynthesis.

Symbol	Enzyme	Number	Sequence ID
*FatA*	Acyl-ACP thioesterase A	2	CL750.Contig1_All, Unigene9739_All
*FatB*	Acyl-ACP thioesterase B	1	CL9346.Contig1_All
*ACC*	Acetyl-CoA carboxylase	6	CL4333.Contig5_All, Unigene822_All, CL5288.Contig2_All, CL1365.Contig6_All, Unigene5615_All, CL1365.Contig3_All
*EAR*	Enoyl-ACP reductase	2	CL8114.Contig4_All, CL8114.Contig3_All
*KAR*	Ketoacyl-ACP reductase	1	Unigene21713_All
*KASI*	Ketoacyl-ACP synthase I	2	CL357.Contig1_All, CL247.Contig2_All
*KAS II*	Ketoacyl-ACP synthase II	5	CL9770.Contig1_All, CL9770.Contig2_All, CL6776.Contig1_All, CL6776.Contig2_All, CL2630.Contig4_All
*KAS III*	Ketoacyl-ACP synthase III	2	CL8481.Contig3_All, CL8481.Contig2_All
*KAS IV*	Ketoacyl-ACP synthase IV	1	CL9211.Contig2_All
*MAT*	Malonyl-CoA ACP transacyclase	2	CL9289.Contig4_All, CL9289.Contig1_All
*FAD2*	Oleoyl-ACP desaturase	5	Unigene9738_All, Unigene14717_All, CL762.Contig1_All, CL4265.Contig1_All, CL4265.Contig2_All
*FAD3*	Oleoyl-ACP desaturase	1	CL8454.Contig1_All
*FAD7*	Oleoyl-ACP desaturase	2	Unigene12140_All, Unigene12140_All
*SAD*	Stearoyl-ACP desaturase	2	Unigene18712_All, CL1502.Contig3_All

**Fig 9:**
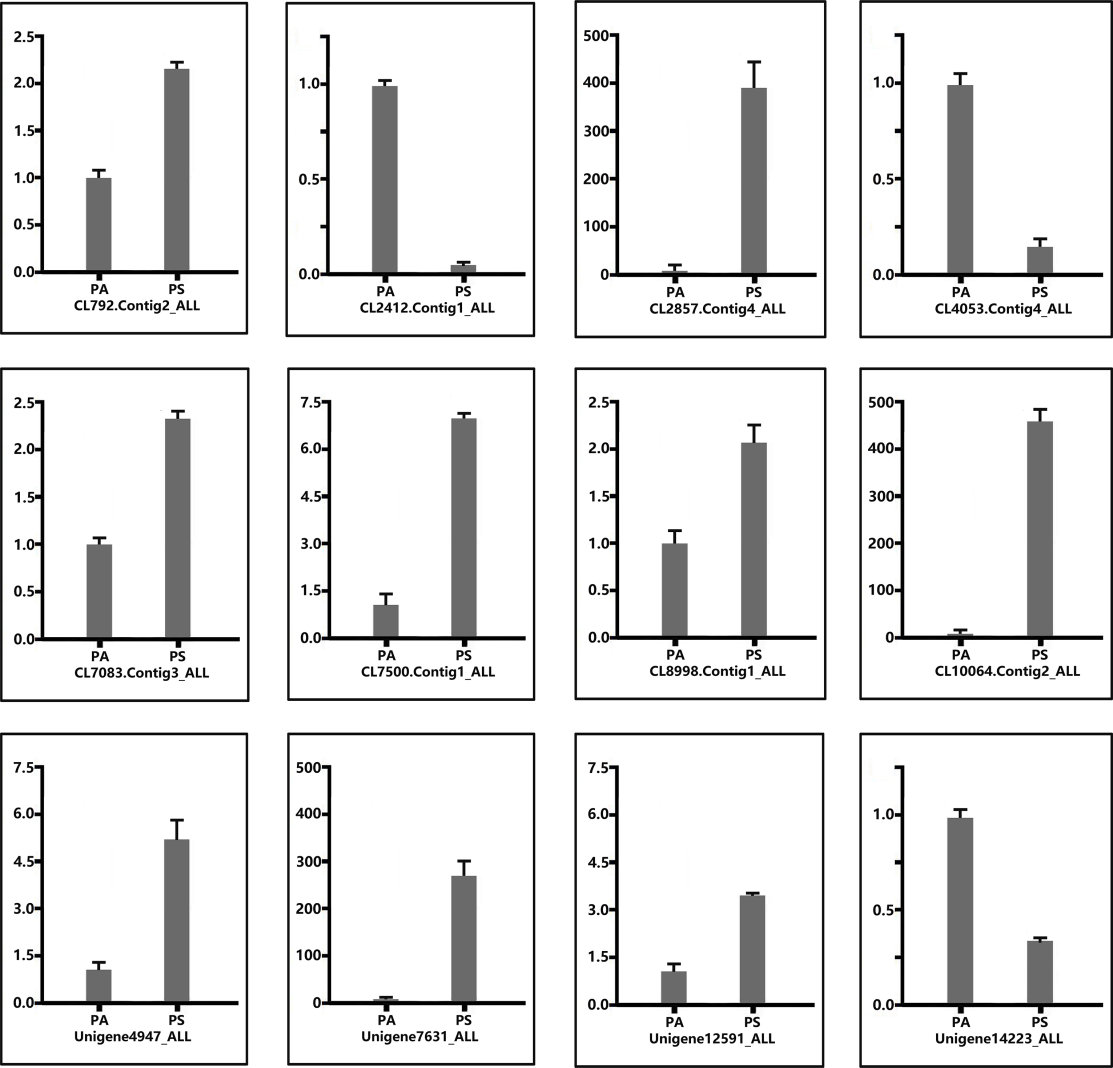
Quantitative real time PCR (qPCR) analysis of the expression of several key genes involved in TAG biosynthesis. *GAPDH* (Unigene14735_All) was used as the internal reference. The error bars represent SE (n = 3). PA, initiate stage; PS, fast oil accumulation stage.

Triacylglycerol acid and oleosins are the main energy stocks in the seeds. In the pathway of TAG assembly, there are three unigenes encoding glycerol-3-phosphate acyltransferase (GPAT, which catalyzes the first step of TAG biosynthesis), five unigenes for acyl-CoA: diacylglycerol acyltransferase (DGAT, which transfer the acyl group to the 1, 2-diacylglycerol to form TAG), and four unigenes for NAD-dependent glycerol-3-phosphate dehydrogenase (GPDH, which catalyzes sn-glycerol 3-phosphate, an initial substrate for TAG synthesis). The results showed that all the TAG biosynthesis unigenes were substantially expressed at the fast oil accumulation stage, indicating the pivotal role of these unigenes playing in TAG synthesis (Table 5). Among these genes related to TAG synthesis, 12 genes were randomly selected and their gene expression by RNA seq was further validated by quantitative real time PCR (Fig 9). In the plant cell, TAG is usually stored in the oil bodies, of which is surrounded by oleosin or steroleosin in the seeds. Thus the fast accumulation of TAG is also accompanied with an increased level of oleosin proteins. From the libraries of pecan seeds at different developing stages, we identified 10 unigenes encoding oleosin proteins. The expression of these oleosin genes was very high at the fast oil accumulation stage, which is also in accord with the expression pattern of fatty acid and TAG synthesis genes.

**Table 5:** Unigenes related to TAG biosynthesis.

Symbol	Enzyme	Number	Sequence ID
*LAT*	1-Acylglycerol-3-phosphate-O-acyltransferase	1	Unigene20649_All
*DGAT*	Acyl-CoA:diacylglycerol acyltransferase	5	Unigene4947_All, CL7779.Contig1_All, CL4053.Contig5_All, Unigene20649_All, CL4053.Contig4_All
*GPAT*	Glycerol-3-phosphate acyltransferase	3	Unigene18145_All, CL5515.Contig1_All, CL5515.Contig2_All
*PDAT*	Phospholipid:diacylglycerol acyltransferase	8	Unigene16582_All, Unigene190_All, CL6106.Contig1_All, Unigene21609_All, Unigene12591_All, CL144.Contig5_All, CL792.Contig2_All, CL2412.Contig1_All
*OLE*	Oleosin	10	CL10064.Contig2_All, CL2857.Contig2_All, Unigene1979_All, Unigene7965_All, CL10064.Contig1_All, Unigene7631_All, Unigene19032_All, CL2857.Contig1_All, CL2857.Contig3_All, CL2857.Contig4_All
*GPDH*	NAD-dependent glycerol-3-phosphate dehydrogenase	1	CL7500.Contig1_All, Unigene14223_All, CL7083.Contig3_All, CL8998.Contig1_All

### Identification of unigenes encoding allergens in the pecan seeds

Although pecan nuts have been enjoyed safely by millions of consumers, it was also found that many people are allergic to the allergens in the seeds. In pecan seeds, the main allergens represent a class of pecan proteins [34]. The food allergens in the seeds were shown to be stable, even under in vitro proteolysis, whereas the non-allergenic proteins can be fast digested under the same conditions [35]. It was important to minimize the activity of the allergens before the pecan seeds were processed to food. Characterization of the allergen proteins in pecan seeds would be helpful in practice to decrease the allergen content. In the molecular level, the identification of the key genes encoding the allergen proteins would be helpful to generate the allergen-free pecan nuts by knocking out these allergen genes using the genome editing technology. In the transcriptome library of pecan seeds collected from differential developing stages, we identified 76 unigenes that could encode the allergen or allergen-related proteins. Among the 30 most abundant unigenes at the fast oil accumulation stage (PS), two unigenes (Unigene679_All and CL3605.Contig2_All) separately encoding allergen I1 and major latex allergen Hev b5 were identified (Table 3). The high expression level of allergen-encoded genes at PS stage indicated a high accumulation of allergen proteins during the ripping (should this be ripening?) of the pecan seeds. Three other unigenes, CL7385.Contig1_All, CL5380.Contig4_All and CL3308.Contig2_All, which separately encode Allergen Hev b 8.0201, Major pollen allergen Pla l 1 and Allergen Pru du 3.02, were found to be highly expressed at PA stage, whereas down-regulated at PS stage (Table 6). The identification of allergen-encoded unigenes in this work would provide potential molecular targets for generating the allergen-free pecan cultivars.

**Table 6:** Identification of unigenes coding allergens.

Gene ID	Gene product	Length (bp)	PA-MEAN (RPKM)	PS-MEAN (RPKM)
Unigene679_All	Allergen I1	884	764.47	89332.9
CL3605.Contig2_All	Major latex allergen Hev b 5	456	3167.13	3802.77
CL7385.Contig1_All	Allergen Hev b 8.0201	867	1414.83	490.66
CL5380.Contig4_All	Major pollen allergen Pla l 1	823	831.94	207.45
CL3308.Contig2_All	Allergen Pru du 3.02	888	863.98	110.13
CL1748.Contig3_All	Allergen-related	1312	47.47	46.03
CL7363.Contig1_All	Major pollen allergen Lol p 11	795	56.56	25.05
CL8765.Contig2_All	Allergen Pru p 2.04	1230	22.35	11.03
Unigene11901_All	Pollen Ole e 1 family allergen	644	2.48	5.64
Unigene13131_All	Pollen Ole e 1 family allergen	427	2.48	1.95
CL6645.Contig2_All	Major pollen allergen Bet v 1-D/H	548	3.51	1.22
CL3530.Contig1_All	Major pollen allergen Ory s 1	1133	45.12	0

## Conclusions

In this work, we report a comprehensive dataset by high-throughput sequencing technology for pecan. Transcriptome analyses of pecan seeds at different developing stages revealed 82,155 unigenes. Further analyses from two developmental stages (the initial stage, and the fast oil accumulation stage) of pecan nuts showed abundant unigenes differentially expressed between these two libraries, with 5580 unigenes up-regulated, and 7745 down-regulated. These identified unigenes could be involved in fatty acid biosynthesis and degradation, TAG biosynthesis, and some other developing aspects. Given the economic significance of pecan as an important resource of edible unsaturated oil, pecan still needs agronomic improvement, such as increasing the seed oil content, generating an allergen free pecan nut, and so on. To achieve this goal, the identification of the genes that are involved in regulating these biological processes is very important. The transcriptome dataset provided in this work would be helpful for the molecular breeding of pecan.

## Data archiving statement

The authors confirm that all data underlying the findings are fully available without restriction. All the clean reads have been submitted to the sequence read archive (SRA) at NCBI with the accession number PRJNA431045.

## Competing interests

The authors declare that we have no competing interests.

## Supporting information

S1 Table List of primers used for qPCR analysis in this study. (DOCX)

S2 Table Summary of functional annotation result. (DOCX)
