# Supplementary material for: Transcriptome analysis of pecan seeds at different developing stages and identification of key genes involved in lipid metabolism
Source: PLoS One. 2018 Apr 25;13(4):e0195913. doi: 10.1371/journal.pone.0195913 (PMC5919011; doi:10.1371/journal.pone.0195913)
Supplement: S1 Table — (DOCX) [file pone.0195913.s001.docx]

**Table S1 List of primers used for qPCR analysis in this study**

| **Gene ID** | **Direction** | **Sequence** | **Purpose** |
| --- | --- | --- | --- |
| Unigene14735_All | Forward | ACATCAAGGCTGCTATTAAGGAAGAGTC | qPCR |
| Unigene14735_All | Backward | GTCATACCAGGTCACGAGTTTCACAA | qPCR |
| CL792.Contig2_All | Forward | CAATCACCTCACTGCTCTT | qPCR |
| CL792.Contig2_All | Backward | TCTGAACTACTCCTGTGTTG | qPCR |
| CL2412.Contig1_All | Forward | CAGGAGTTCTCGTTGGCGATCAC | qPCR |
| CL2412.Contig1_All | Backward | AGTTCTTGGCACCATGTAACCTTCC | qPCR |
| CL2857.Contig4_All | Forward | AATCGACCAGGCACGCATGAAG | qPCR |
| CL2857.Contig4_All | Backward | TGGAGTTGGAGGAGGAGGAGGA | qPCR |
| CL4053.Contig4_All | Forward | CGAAGAGCATCAATGTGACACCAGA | qPCR |
| CL4053.Contig4_All | Backward | GTTGCCTTACACTCCCGATATTCCC | qPCR |
| CL7083.Contig3_All | Forward | TAGGCTCAGGCATTTGGTCGTAAAC | qPCR |
| CL7083.Contig3_All | Backward | CATGATCGTCGCTGGCACTCTC | qPCR |
| CL7500.Contig1_All | Forward | CCGAAGACTCAGGCTGGTTGATATG | qPCR |
| CL7500.Contig1_All | Backward | ATGACAGCAAGGATACGAAGCAGTG | qPCR |
| CL8998.Contig1_All | Forward | CTACAAGGATCTCAAGGCACTCAACC | qPCR |
| CL8998.Contig1_All | Backward | AGGCTCAATCTCGCTGCATTCAC | qPCR |
| CL10064.Contig2_All | Forward | TCCCTCCTGCTACTCTCTGGTTTG | qPCR |
| CL10064.Contig2_All | Backward | TCACCGCCGCTACTCCGAAT | qPCR |
| Unigene4947_All | Forward | CGCAATCTTCTTACCTCCGTCGTT | qPCR |
| Unigene4947_All | Backward | ACTCGGCTAGAGAATCGGTCACAT | qPCR |
| Unigene7631_All | Forward | ATTGGTGCTACGCTCCTAATCTTGTC | qPCR |
| Unigene7631_All | Backward | GGCAGCAACCAGGAATAAGACTATACC | qPCR |
| Unigene12591_All | Forward | ACAGTGAGGTAGGAAAGGGCTCTATC | qPCR |
| Unigene12591_All | Backward | CATGGATCGCCAAGATGAAGGTGAA | qPCR |
| Unigene14223_All | Forward | CCAATGAACTTGCTGACGCTGATG | qPCR |
| Unigene14223_All | Backward | GGAGTAGTCTCTTGTCCACCACCTT | qPCR |
