# Supplementary material for: Transcriptome analysis of pecan seeds at different developing stages and identification of key genes involved in lipid metabolism
Source: PLoS One. 2018 Apr 25;13(4):e0195913. doi: 10.1371/journal.pone.0195913 (PMC5919011; doi:10.1371/journal.pone.0195913)
Supplement: S2 Table — (DOCX) [file pone.0195913.s002.docx]

**Table S2 Summary of functional annotation result**

| Values | Nr | Nt | Swissprot | KEGG | COG | Interpro | GO | Overall |
| --- | --- | --- | --- | --- | --- | --- | --- | --- |
| Number | 56,634 | 56,391 | 39,683 | 43,881 | 22,949 | 39,613 | 32,499 | 61,938 |
| Percentage | 68.94% | 68.64% | 48.30% | 53.41% | 27.93% | 48.22% | 39.56% | 75.39% |
